# Metabolite Quantification by Fourier Transform Infrared Spectroscopy in Diatoms: Proof of Concept on *Phaeodactylum tricornutum*

**DOI:** 10.3389/fpls.2021.756421

**Published:** 2021-11-10

**Authors:** Matteo Scarsini, Adrien Thurotte, Brigitte Veidl, Frederic Amiard, Frederick Niepceron, Myriam Badawi, Fabienne Lagarde, Benoît Schoefs, Justine Marchand

**Affiliations:** ^1^Mer Molécules Santé, Le Mans University, IUML-FR 3473 CNRS, Le Mans, France; ^2^Institute of Molecular Biosciences, Goethe University Frankfurt, Frankfurt, Germany; ^3^UMR CNRS 6283 Institut des Molécules et des Matériaux du Mans, Le Mans University, Le Mans, France

**Keywords:** FTIR spectroscopy, *Phaeodactylum tricornutum* Bohlin, macromolecules quantification, algal physiology, lipid droplets, phytoplankton

## Abstract

Diatoms are feedstock for the production of sustainable biocommodities, including biofuel. The biochemical characterization of newly isolated or genetically modified strains is seminal to identify the strains that display interesting features for both research and industrial applications. Biochemical quantification of organic macromolecules cellular quotas are time-consuming methodologies which often require large amount of biological sample. Vibrational spectroscopy is an essential tool applied in several fields of research. A Fourier transform infrared (FTIR) microscopy-based imaging protocol was developed for the simultaneous cellular quota quantification of lipids, carbohydrates, and proteins of the diatom *Phaeodactylum tricornutum*. The low amount of sample required for the quantification allows the high throughput quantification on small volume cultures. A proof of concept was performed (1) on nitrogen-starved experimental cultures and (2) on three different *P. tricornutum* wild-type strains. The results are supported by the observation *in situ* of lipid droplets by confocal and brightfield microscopy. The results show that major differences exist in the regulation of lipid metabolism between ecotypes of *P. tricornutum*.

## Introduction

The increase of human populations, the reduction of farmland and the expansion of cities contribute to an accelerated depletion of natural resources to a point that finding alternative sources of natural resources, including biomolecules, is becoming crucial. Microalgae are a promising feedstock for the production of biomolecules belonging to the major organic macromolecules classes (Mimouni et al., 2012) while utilizing sun energy through the photosynthetic process (Scarsini et al., 2019). In addition, microalga culture does not require farmland, reducing the competition for space with other farm productions (Gordon et al., 2019). Unfortunately, the current knowledge on microalgae does not allow the development of biotechnological sustainable processes from the ecological and financial point of views. Several bottlenecks need to be addressed before establishing microalgae as efficient cell factories (Vinayak et al., 2015). Obtaining appropriate strains dedicated to a specific production is one of the major challenges. To reach this goal, two strategies may be applied: (1) the exploration of microalgal biodiversity to find appropriate strains with high industrial application potential and (2) the genetic modifications of wild-type microalgal strains to improve their productivity. The efficient and fast selection of natural and genetically improved strains is thus seminal to identify the strains that perform better when cultured. The development of large-scale screening techniques is a challenge. In particular, the identification of cell lines, which possess the phenotype of interest among a mutagenized population, can be a particularly tedious process for which it may be necessary to analyze hundreds or even thousands of transformants.

High throughput methods for microalgal cultivation in small volumes have been developed since a long time (Heinemann and Paulsen, 1999). More recently, Van Wagenen et al. (2014) described a methodology that allows the cultivation of microalgae in small volume multi-well plates with results comparable to a photobioreactor cultivation. However, the development of a macromolecule quantification screening method which does not require large sampling volumes is fundamental since conventional biochemical analyses require relatively high amounts of culture sample.

Fourier transform infrared spectroscopy (FTIR) is a widespread vibrational spectroscopy technique that can provide a chemical fingerprint of samples (either solid, liquid, or gas) with a wide range of applications. In the field of microalgae, FTIR was used to confirm the metabolic plasticity that microalgae display in response to environmental stress such as nutrient stress (Giordano et al., 2001; Heraud et al., 2005; Stehfest et al., 2005; Sigee et al., 2007; Dean et al., 2008; Dean et al., 2010; Wagner et al., 2010; Bajhaiya et al., 2015; Driver et al., 2015; Giordano et al., 2017; Pogorzelec et al., 2017; Cointet et al., 2019). However, most of these studies focused on freshwater microalgae while only few FTIR spectroscopy analyses have been performed on marine microalgae, including diatoms (Giordano et al., 2017; Pogorzelec et al., 2017; Cointet et al., 2019). Among diatoms, *Phaeodactylum tricornutum* is a popular widely used model species, for both physiology studies and genetic engineering (Heydarizadeh et al., 2014, 2017, 2019; Bowler and Falciatore, 2019; Butler et al., 2020). Ten different *P. tricornutum* ecotypes have been isolated around the world, differing on both genotypic and phenotypic levels (Martino et al., 2007; Rastogi et al., 2020). The genetic modification tools available for this species make it possible to create mutants relatively easily (*e.g.*, Nymark et al., 2016; Serif et al., 2018; Stukenberg et al., 2018; Hu and Pan, 2020).

This manuscript reports a high-throughput phenotypic microalgal screening methodology based on FTIR-coupled microscopy to quantify lipids, proteins and carbohydrates cell contents in different growth conditions and different ecotypes or strains of the diatom *P. tricornutum*.

## Materials and Methods

### Microalgal Strains and Culture Conditions

Two ecotypes were used in this study: Pt4 (UTEX 646) and Pt1 (CCMP 2561). Two Pt1 strains were studied, coming from geographically different laboratories: Pt1-Wuhan, kindly provided by Pr H. Hu from the Center for Algal Biology and Applied Research from the Chinese Academy of Sciences at Wuhan (China), and Pt1-Paris kindly provided by Dr. A. Falciatore from the Laboratoire de Biologie du Chloroplaste et Perception de la Lumière chez les Microalgues, Institut de Biologie Physico-Chimique (IBPC, Paris, France).

Both *P. tricornutum* ecotypes were cultured in f/2 medium (Guillard, 1975) supplemented with bicarbonate (14.3 mM NaHCO_3_). Precultures and experimental cultures were constantly maintained at 21 ± 1°C and 150 μmol m^– 2^ s^– 1^. Culture axenicity was checked by plating a drop of culture onto LB medium and f/2 medium supplemented with 0.5% bacto-peptone and 0.1% yeast extract, both incubated in the dark at 21°C for at least 7 days to check for eventual bacterial presence. *P. tricornutum* was cultivated in cell culture flasks (T-25 and T-75 according to the experiment requirement, Sarstedt). To obtain *P. tricornutum* cells (Pt4) enriched in lipids, microalgae were cultivated in nitrogen (N)-depleted f/2 medium (*i.e.* [NO_3_^–^ ]=0). To validate the FTIR methodology, a N-starvation experiment was conducted on Pt4 in a 1 L laboratory scale photobioreactor (PBR) (FM150, Photon System Instruments, Czechia) operated in turbidostat mode (∼3.5 × 10^6^ cells ml^– 1^) as described in Scarsini (2021). Briefly, the culture was continuously supplied with 1.2 ml min^– 1^ of a mixture air/CO_2_ (2,000 ppm CO_2_) at 21°C and 150 μmol photons s^– 1^ m^– 2^ (white LED panel). The culture was initially exposed in non-limited conditions (5 days) and then fed with f/2 medium containing 0.15 mM NaNO_3_ for 20 days. Samplings along the starvation were performed.

A N-starvation experiment (NO_3_^–^ depleted f/2 medium) was finally conducted on Pt1-Wuhan, Pt1-Paris and Pt4: exponential cultures were transferred from N-repleted to N-depleted medium for 18 days in flasks in the same conditions than described above (temperature, light). Samplings were performed along the starvation at 0, 6, 10, and 18 days.

### Cell Counting and Growth

Cell density was determined using a Neubauer hemacytometer (Hausser Scientific, Horsham, PA, United States). For the comparison between ecotypes, cell densities were determined using a double beam spectrophotometer (Lambda 25, Perkin Elmer). Optical density (OD) was measured at 735 nm and calibration curves prepared for each strain and each condition in order to associate OD to cell density. Calibration curves are reported in Supplementary Figure 1A. Determination of growth rates was obtained using the software CurveExpert Basic^1^ through a logistic model fitting (Bolzmann).

### Fourier Transform Infrared Spectroscopy

Samples were centrifugated at 3,000×*g* for 10 min, washed twice with a NaCl solution (1,032 mOsmol), vacuum dried for 5 min and stored at –20°C until use. Samples were resuspended in ultrapure water in order to obtain the desired number of cells ml^– 1^. One μl of the suspension was deposited on a silicon plate and dried at 37°C for 15 min. The silicon plate was previously carefully washed with isopropanol (Fisher Scientific, >99.5%) and dried. Three biological samples (three drops) were considered. Infrared (IR) spectra were collected at the Vibrational Spectroscopy platform of the IMMM (Le Mans University) using a FTIR-microscope Spotlight 200i (Perkin Elmer) controlled by the Spectrum^TM^ software (Perkin Elmer). Spectra were collected from eight randomly distributed spots on the drop avoiding the centre (Supplementary Figure 2) with a 4 cm^– 1^ spectral resolution and 32 scans per spectrum in transmission modus. Each spectrum was collected in the range 800–4,000 cm^– 1^ (Supplementary Figure 3A) on a square surface area of 10^4^ μm^2^. A cut-down spectrum in the range 800–2,000 cm^– 1^ (Supplementary Figure 3B) was also used. For construction of the baseline, the spectrum was divided into n ranges determined from a convex hull of the spectrum. The minimum Y value of each range was determined. Connecting the minima with spline lines creates the baseline. Starting from “below,” a rubber band was stretched over this curve (Supplementary Figure 3C). The baseline points that do not lie on the rubber band were discarded. The baseline was subtracted from the raw data to yield the flattened spectrum (Supplementary Figure 3D; Shen et al., 2018). To normalize spectra, the Standard Normal Variate (SNV) method was used (Barnes et al., 1989). The SNV is performed first calculating the average intensity value and subsequently subtracting this value from each spectrum (Supplementary Figure 3E). Then, the sum of the squared intensities was calculated, and the spectrum divided by the square root of this sum (the standard deviation). After SNV, each spectrum has a mean of 0 and a standard deviation of 1 (offset correction) (Supplementary Figure 3F). HyperSpec version 0.99^2^, prospectr version 0.2.1^3^ (Barnes et al., 1989) and base version 4.0.4 packages were used to perform rubber-band baseline correction, normalization and offset correction, respectively. All packages were compiled with R version 4.1.0. Analyses were performed using the arithmetic mean spectra resulting from three biological replicates (three drops per sample) themselves resulting from eight technical replicates (eight spectra per drop).

### Metabolite Quantifications

Sample were centrifugated at 3,000×*g* for 10 min, washed twice with a NaCl solution (1,032 mOsmol) and stored at –20°C until use. Neutral lipid cell quota (called Q_*Lipids*_) was quantified by Nile Red staining according to Huang et al. (2019). Room temperature fluorescence spectra were recorded with the Perkin Elmer LS-55 (PerkinElmer^®^, Excitation wavelength: 530 nm, Emission: 545–800 nm, slit: 5 nm). Dimethyl sulfoxide-diluted glyceryl trioleate (Sigma-Aldrich T7140-500MG, purity ≥ 99%) was used for calibration (Supplementary Figure 4A). Soluble proteins and total carbohydrates cell quota (denoted as Q_*Proteins*_ and Q_*Carbohydrates*_ respectively) were measured according to Heydarizadeh et al. (2019). Colorimetric analyses were performed using the Lambda-25 spectrophotometer (PerkinElmer^®^) at 595 and 485 nm (slit: 5 nm) for proteins and carbohydrates, respectively. Bovine serum albumin (Sigma-Aldrich, purity ≥98%) and glucose were used for proteins and carbohydrates quantification calibrations respectively (Supplementary Figures 4B,C). Photosynthetic pigments were extracted using acetone (Merck, ≥99.5%) according to Heydarizadeh et al. (2017). Absorbance spectra were recorded between 400 and 800 nm (Perkin Elmer Lambda-25 spectrophotometer) and Chl *a*, Chl c and total carotenoids concentrations calculated according to equations from Heydarizadeh et al. (2017).

### Brightfield Microscopy for the Estimation of Cell Size

Pictures were collected with an Olympus CX23 brightfield microscope (objective Olympus 40X) equipped with a ISH300 digital camera (Tucsen Photonics, China). Fiji software^4^ (ImageJ v1.53c) was used to measure cell lengths and widths of the different *P. tricornutum* ecotypes and strains.

### Confocal Microscopy for the Estimation of Lipid Droplet Volumes

Samples of Pt1 strains and Pt4 were used for imaging using laser scanning confocal microscopy (LSCM). Lipid droplets (LDs) were stained using BODIPY 505/515 (Invitrogen Molecular Probes, Inc., CA, United States), a lipophilic dye with improved properties for confocal imaging. In comparison to Nile Red, the BODIPY 505/515 has a narrower emission spectrum allowing fluorescence enhancement of LDs (Cooper et al., 2010; Govender et al., 2012). One ml of culture (5 × 10^6^ cells ml^−1^) was treated with 50 μl of DMSO and stained during 10 min with 1 μl of BODIPY 5 mM. The number of cells analyzed for each replicate ranged from 50 to 180 cells (three biological replicates were considered). Three-dimensional projections of LD were performed capturing stack pictures of the sample (400 nm Z resolution) at the Confocal Microscopy platform of the IMMM (Le Mans University) using a LSM800 laser scanning confocal microscope (Carl Zeiss Microscopy GmbH, Germany). All images were acquired using a 63× objective (LCI Plan-Neofluar with numerical aperture of 1.3 water immersion objective DIC M27) with 0.5 as scan zoom. Z sectional images were captured using the 488 and 561 nm line excitation lasers (at respectively 4.5 and 5% laser intensities) for BODIPY and Chl *a* autofluorescence respectively. The BODIPY emission was detected between 410 and 546 nm and Chl *a* autofluorescence between 650 and 700 nm. Z-slice step size was 0.33 μm slice^– 1^ increments. Image dimensions were 2,297 × 2,297 pixels with sampling speed of 0.38 μs pixel^– 1^ and size of 0.073 μm pixel^– 1^. Photomultiplier tubes gain were manually adjusted to provide optimal brightness and resolution. Fluorescent images were merged and colored using the Zen Blue software (Zeiss). Fiji software^4^ (ImageJ v1.53c) was used to estimate the volume of LDs. Stacks from LSCM images containing only the fluorescence channel were converted to a Z-projection. The diameter of each LD was measured manually and used to calculate the volume of a sphere (assuming that all LDs are spherical).

### Statistics

Linear and non-linear fitting analyses were performed with the Curve Expert software^1^. Analyses of variance (ANOVA) were performed using a python script exploiting scipy (version 1.5.2), statsmodels (version 0.12.0), and bioinfokit (version 2.0.3).

## Results and Discussion

A high-throughput sensitive methodology based on FTIR was designed and evaluated on the marine diatom *P. tricornutum*. It allowed to assess carbon allocation changes in response to N-starvation.

### Typical Infrared Spectra of *Phaeodactylum tricornutum*

The functional groups attached to the different organic macromolecules absorb IR radiation at specific wavelengths, generating a complex spectrum considered as a fingerprint of the cellular composition (Movasaghi et al., 2008; Wagner et al., 2010). For a suitable exploitation, three to four steps are necessary: (1) the FTIR full raw spectrum (800–4,000 cm^– 1^) needs to be processed (Supplementary Figure 3A); (2) a cut-down of the spectrum can be further performed keeping only wavenumbers included in 800–2,000 cm^– 1^ to avoid the spectral perturbation generated by the large water peak (Supplementary Figure 3B); (3) a baseline correction either on the entire spectrum or the cut-down spectrum (Supplementary Figures 3C,D); and (4) a normalization procedure can be performed (Supplementary Figures 3E,F). FTIR spectra often exhibit baseline offset and curvilinear trend caused by changes in optical path length and light scattering. Baseline removal aims at resetting all spectra on a common baseline (Sandak et al., 2016; Shen et al., 2018). The normalization procedure is carried out in order to uniform the information from spectra and to correct spectra for changes in optical path length and light scattering. This process can be particularly important when dealing with inhomogeneous samples which is the case in microalgae cultures (Sandak et al., 2016). SNV normalization was used to transform each measured spectrum into a signal with a mean of 0 and a variance of 1 (Barnes et al., 1989): the mean value of the spectrum (the offset) (Supplementary Figure 3E) is subtracted to each absorbance value, which is subsequently divided by its respective raw standard deviation (Supplementary Figure 4F). The data obtained in this study are presented in two different ways: (1) baseline-corrected only and (2) baseline-corrected followed by a SNV normalization.

**FIGURE 1:**
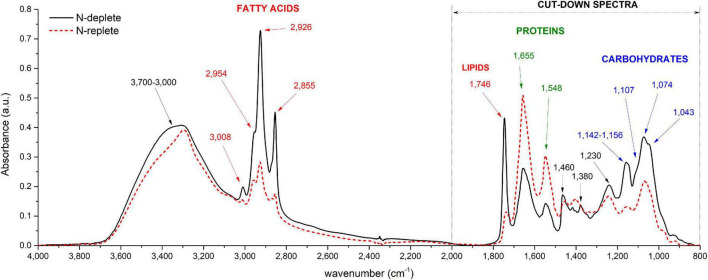
Typical FTIR spectra recorded from *P. tricornutum* (Pt4) in two different conditions: N-replete (blue line) and N-deplete (red line). Major band assignments are indicated by arrows and defined in Table 1.

Examples of baseline-corrected spectra obtained from a culture sample of *P. tricornutum* in two different N availability conditions are reported in Figure 1. Different peak intensities at particular wavenumbers are observed depending on the N availability in which cells have been grown (Figure 1). Two spectral ranges are particularly affected: (1) 800 to 2,000 cm^– 1^ and (2) 2,800 to 3,100 cm^– 1^. Strong absorption bands at approximately 1,043, 1,074, 1,107, 1,142, and 1,156 cm^– 1^ observed mostly in N-depleted condition correspond to the vibration of C–O bonds of polysaccharides (Table 1; Giordano et al., 2001; Mayers et al., 2013). Absorption bands observed at 1,548 and 1,655 cm^−1^ correspond to the vibration of the N-H, C-N and C=O of amide groups belonging to proteins (Table 1). The stretching C=O bonds of esters of lipids and fatty acids are found at 1,746 cm^– 1^ while those at 2,855, 2,926, and 2,954 cm^– 1^ correspond to the symmetric and asymmetric stretching of CH_2_ and CH_3_ methyl and methylene groups of fatty acids (Table 1). As demonstrated by Wagner et al. (2010), absorbance peak height can be used for macromolecules quantification instead of peak integral. On an overall analysis, the N-starvation induces an increase in lipid and carbohydrate cell quotas as well as a decrease in protein cell quota in comparison to N-replete conditions (Figure 1), as observed in literature through conventional biochemical analyses (Huang et al., 2019).

**TABLE 1:** Band assignment and functional groups of a typical spectrum of *P. tricornutum*.

Wavenumber (cm^– 1^)	Assignment
3,700–3,000	*_*V*_*O-H of water, *_*V*_*N-H of amide, *_*V*_*C-O of carbohydrates
3,008	*_*V*_*C-H of C=CH– chains of lipids
2,954	*_*Vas*_*CH_3_ of methyl groups
2,926	*_*Vas*_*CH_2_ of methylene groups
2,855	*_*V*_*CH_2_ and *_*V*_*CH_3_ of methyl and methylene groups
1,746	*_*V*_*C=O ester of lipids and fatty acids
1,655	*_*V*_*C=O of proteins (Amide I)
1,548	*_δ_*N-H and *_*V*_*C–N of proteins (Amide II)
1,460	*_δ*as*_*CH_2_ and *_δ*as*_*CH_3_ of methyl and methylene groups
1,380	*_δ_*CH_2_ and _δ_CH_3_ from proteins and *_δ_*C–O from carboxylic groups
1,230	*_*Vas*_*P=O from phosphodiester of nucleic acids and phospholipids
1,200–900	*_*V*_*C-0-C from polysaccharides
980	P-O-P of Polyphosphates

*Band assignments are taken from references Giordano et al. (2001) and Mayers et al. (2013). *_*V*_*, symmetric stretching; *_*Vas*_*, asymmetrical stretching; *_δ_*, symmetric deformation (bend); *_δ*as*_*, asymmetric deformation (bend).*

### Reproducibility of Fourier Transform Infrared Measurements

Reproducibility, as a major principle of a scientific method, is a key feature of an effective analysis. In previous works (Dean et al., 2010; Wagner et al., 2010; Driver et al., 2015), FTIR was performed on a unique sensor and samples required time to dry, making this technique low throughput. In our work, we used imaging spectroscopy that allows to visualize the sample on which the analysis is performed. Multiple areas can thus be selected to record the IR spectra. This allowed to randomly collect absorbance spectra in different areas of the sample *i.e.* recording technical replicates within the same sample (Supplementary Figure 2), and different samples within the same holder.

The reproducibility among both technical and biological replicates was tested. Reproducibility of the results increases with the number of technical replicates (Supplementary Figure 5A). Indeed, no significant difference (ANOVA, *p*<0.05) was found between the 8 technical replicates for all the wavenumbers studied. However, a higher variability was found (particularly visible for the 1,746 cm^– 1^ peak representing lipids) when less than 4 replicates were considered (Supplementary Figure 5A). The lack of uniformity of the dried drop on the measuring plate can explain the variability of the measurements (Supplementary Figure 2). Eight randomly distributed spots of analyses were set as the optimal compromise between statistical significance, reproducibility and time spent on each sample for the analyses.

Biological replicates consist in samples containing different cells cultivated in the same culture conditions. Six biological replicates (drops A–F; Supplementary Figures 5B,C) were tested in order to evaluate the inter-sample reproducibility. The peak amplitude for each biological replicate is the average of the eight technical replicates. From these analyses, different results were observed according to the different spectra processing steps performed. The single baseline correction (Supplementary Figure 5B) does not allow a complete reproducibility among the different biological replicates: ANOVA tests (*p*<0.05) revealed differences between peak heights among the biological replicates for all the wavenumbers considered (*p*<0.05). Performing SNV normalization on baseline-corrected spectra (Supplementary Figure 5C) reduces the differences among biological replicates and increases the reproducibility of the experiment (no significant difference among replicates, *p*<0.05). Clearly, standardization of spectra using SNV achieves a scaling effect. This normalization was originally proposed to reduce scattering effects in the spectra but was also proved to be effective in correcting the interference caused by variations in pellet thickness or/and optical path (Razak et al., 2020).

### How Does the Number of Cells Impact the Fourier Transform Infrared Spectroscopy Absorbance?

**FIGURE 2:**
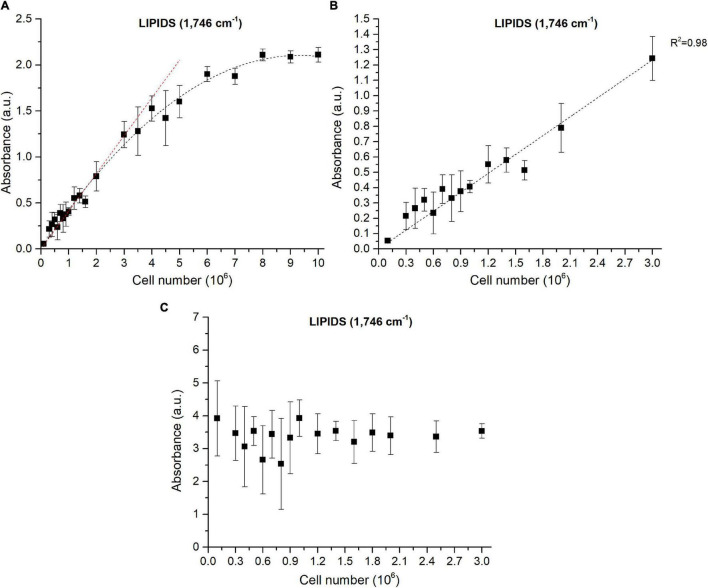
Correlations between FTIR absorbance measured at 1,746 cm^−1^ and the number of cells deposited on the silica plate **(A)** Baseline-corrected spectra considering 0 to 10 million cells, **(B)** Baseline-corrected spectra considering 0 to 3 million cells, **(C)** Baseline-corrected and normalized spectra considering 0 to 3 million cells. The coefficient of determination (R^2^) of the linear regression curve is indicated.

As already reported in literature, sample thickness is a crucial point and can lead to deviations from the Beer-Lambert’s law (Wagner et al., 2010). Cell density is influencing the sample thickness and must thus be taken into account. For a given cell density, the size of the deposited drop can be considered constant since it depends on the force of gravity, the surface tension coefficient and the roughness of the surface of the silica plate (Eid et al., 2018), which are constant in the experiments described in this paper. The diameter of the deposited drop was not impacted during the evaporation of the water as reported previously by Mampallil (2014). To verify the effect of cell density on the experiment reproducibility, FTIR analyses were performed on different cellular densities of Pt4 cells enriched in lipids. Different cell densities were prepared and a drop of each solution deposited on the silica reading plate. As expected, the absorbance increased with cell density and reached a saturation level for densities higher than 6 × 10^6^ cells ml^– 1^ (Figure 2A and Supplementary Figure 6A). The correlation between absorbance and number of cells remained linear under 3 × 10^6^ cells for all the wavenumbers considered (Figure 2B and Supplementary Figure 6B). Figure 2C has been obtained through the SNV normalization procedure to remove the inhomogeneity of the sample (which in this case is the cell density). The graph shows how the replicability at 1,746 cm^– 1^ with a number of cells deposited ranging from 10^6^ to 3 × 10^6^ cells is high. This is also the case for the other wavenumbers, although less pronounced (Supplementary Figure 6C). The instability at lower cellular densities is probably linked to the inhomogeneous distribution of cells inside the drop area (Supplementary Figure 7). The stable values observed with the normalization procedure indicates that depositing a specific number of cells is not necessary when remaining between 10^6^ and 3 × 10^6^ cells. The normalization step corrects sampling and human errors when cell density is determined manually (Malassez or Neubauer hemacytometers). Hence, normalization steps avoid determining cell density of the culture which is, in addition to error prone, time-consuming.

### Correlations Between Fourier Transform Infrared Spectroscopy Absorbance and Biochemical Quantification of Metabolites

**FIGURE 3:**
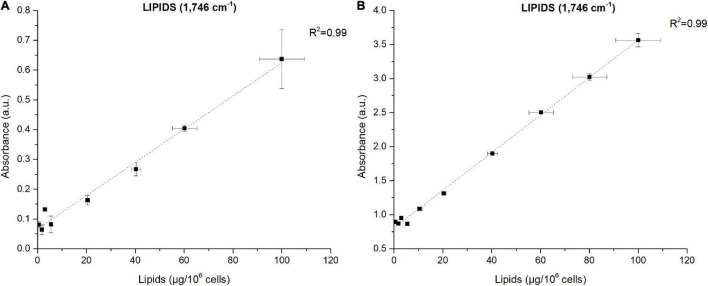
Correlations between FTIR absorbance measured at 1,746 cm^– 1^ and lipids concentrations quantified by fluorescence after Nile-Red staining considering **(A)** baseline-corrected spectra and **(B)** baseline-corrected and normalized spectra. Two million cells were deposited on the silica plate. Coefficients of determination (R^2^) of the linear regression curves are indicated.

The power of FTIR spectroscopy is the quantification at one glance of the three major macromolecular pools (Q_*Lipids*_, Q_*Proteins*_ and Q_*Carbohydrates*_). In literature, both external or internal references have been exploited for their quantification. For example, Wagner et al. (2010) used glucose, glycerol tripalmitate and bovine serum albumin as external references for quantification calibration. However, a biological sample is a very complex matrix containing a mix of molecules, the interactions of which are reflected in the vibrational spectrum (Larkin, 2011). Exploiting external references for the generation of calibration curves could introduce a bias since molecular interactions are not correctly reproduced. This work proposes a different approach: the use of biological samples of *P. tricornutum* as standards. To generate the different standards, a serial dilution of a N-starved Pt4 culture with a N-repleted Pt4 culture was performed to provide a large range of values for lipid, protein, and carbohydrate cell quotas. For each serial dilution, Q_*Lipids*_, Q_*Proteins*_, and Q_*Carbohydrates*_ were determined through conventional biochemical assays. Having determined the effective cell quota and to be in the linear range of the Beer-Lambert’s law, samples of 2 × 10^6^ cells from the different serial dilutions were deposited on the silica plate to perform the FTIR analyses described above. Strong linear correlations were obtained between biochemically quantified lipids and the absorbance at 1,746 cm^– 1^ either after baseline correction (Figure 3A) or normalization (Figure 3B). Correlations for the other wavenumbers are found in Supplementary Figure 8 and regression equations after baseline correction and/or normalization indicated in Table 2.

**TABLE 2:** Regression equations and coefficients of determination (R^2^) of the correlations between FTIR absorbance measured at the different wavenumbers and standard biochemical quantifications considering either baseline-corrected spectra or baseline and normalized-corrected spectra performed on Pt4.

		Baseline spectra		Baseline and Normalized spectra	
Assignments	Wavenumber (cm^– 1^)	Regression equations	*R* ^2^	Regression equations	*R* ^2^
Carbohydrates	1,043	y=0.054x – 0.012	0.90	y=0.054x – 0.012	0.97
	1,074	y=0.057x + 0.007	0.87	y=0.057x + 0.007	0.97
	1,107	y=0.043x – 0.02	0.89	y=0.043x – 0.02	0.98
	1,142	y=0.044x – 0.04	0.90	y=0.044x – 0.04	0.98
	1,156	y=0.048x – 0.05	0.91	y=0.048x – 0.05	0.98
Proteins	1,548	y=0.061x + 0.08	0.78	y=0.061x + 0.08	0.96
	1,655	y=0.085x + 0.2	0.68	y=0.085x + 0.2	0.96
Lipids	1,746	y=0.0056x + 0.07	0.99	y=0.0056x + 0.07	0.99
	2,855	y=0.0045x + 0.18	0.91	y=0.0045x + 0.18	0.97
	2,926	y=0.0071x + 0.29	0.93	y=0.0071x + 0.29	0.98
	2,954	y=0.0034x + 0.24	0.81	y=0.0034x + 0.24	0.93

*y: FTIR absorbance measured at the specific wavenumbers (a.u.). x: concentrations of the different metabolites measured (carbohydrates, proteins, or lipids) with standard biochemical methods.*

The results show that the organic macromolecules classes (lipids, proteins, and carbohydrates) can be quantified exploiting the IR absorbance at their specific wavenumber after the generation of proper calibration curves (*i.e.* using biochemical quantification methods). Performing a normalization increases the reliability of the quantification, particularly for proteins.

### Validation of the Fourier Transform Infrared Spectroscopy Methodology Using Data From a N-Starvation Experiment Conducted in a Photobioreactor

**FIGURE 4:**
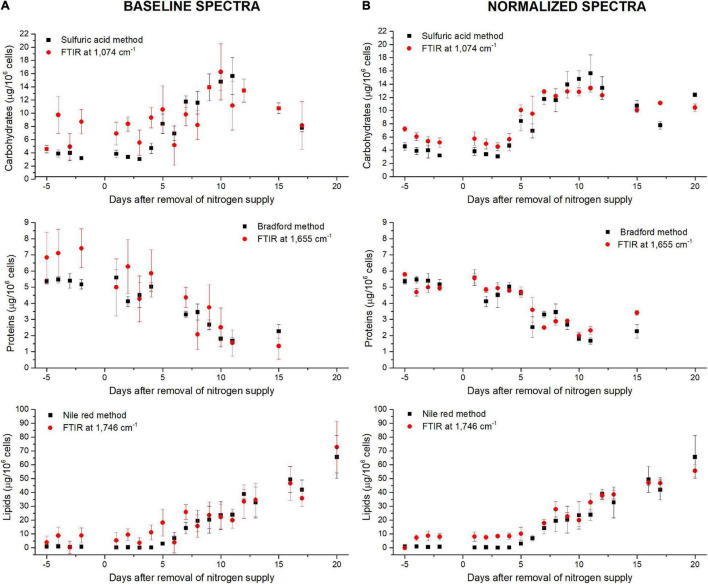
Comparison between standard biochemical and FTIR quantifications along the N-starvation performed in a photobioreactor (Scarsini, 2021) considering **(A)** baseline-corrected spectra or **(B)** baseline and normalized-corrected spectra. FTIR quantifications are performed at 1,074 cm^– 1^ for carbohydrates, 1,655 cm^– 1^ for proteins and 1,746 cm^– 1^ for lipids.

**FIGURE 5:**
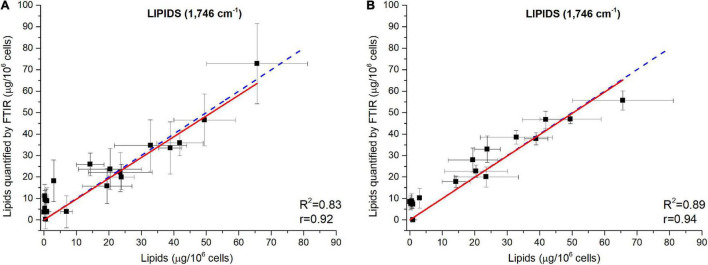
Validation of the FTIR methodology using data from a N-starvation experiment conducted in a photobioreactor (Scarsini, 2021). Correlations between lipids quantified from FTIR absorbance measured at 1,746 cm^– 1^ and lipids concentrations quantified by fluorescence after Nile Red staining considering **(A)** baseline-corrected spectra and **(B)** baseline-corrected and normalized spectra. Two million cells were deposited on the silica plate. Adjusted R^2^ and Pearson’s correlation coefficients of the linear regression curves (r) are indicated on the graphs.

The ability of the FTIR methodology to give results similar to those obtained by traditional methods (Scarsini, 2021) has been tested. Figure 4 shows how both methodologies are comparable. Linear fittings between FTIR quantifications and measured quotas were performed (Figure 5 for lipids at 1,746 cm^– 1^ and Supplementary Figures 9, 10 for carbohydrates, proteins and fatty acids). The blue dotted lines represent theoretical values that must be expected while black squares are the plotted data (biochemical *versus* FTIR quantifications). Linear regressions of the plotted data are reported as well (red continuous lines). Adjusted R^2^ and Pearson’s correlation coefficient of the linear regression curves (r) are higher when normalization is performed (Figure 5A compared to Figure 5B and Supplementary Figure 10 compared to Supplementary Figure 9), confirming that normalizing spectra allows a better quantification. Moreover, the high values of R^2^ (>0.83) and r (>0.92) confirm the coherence between the two quantification methods (excepted for fatty acids wavenumbers).

The relatively lower R^2^ (0.83<R^2^<0.89) obtained for the wavenumber 1,746 cm^– 1^ (Figure 5) could be due to the specificity of the biochemical quantification technique which is designed to quantify neutral lipids while FTIR quantify lipids regardless their class. Scarsini (2021) showed how in N-starvation, cells are principally accumulating neutral lipids through membrane lipid remodeling. The comparison of lipid content estimation through vibrational spectroscopy and a total lipids quantification through another biochemical technique, such as gravimetric analyses (Patel et al., 2019) could potentially reveal a direct linear correlation. The fatty acids measurements (2,855–2,954 cm^– 1^) do not show high correlations, especially when the normalization correction is applied (R^2^<0.43 and r<0.68), probably due to the partial overlapping of these peaks with the large water peak at 3,000–3,700 cm^– 1^. Although better correlations are found for fatty acids with only a baseline correction (R^2^>0.76 and r>0.67), the peak at 1,746 cm^– 1^ is much more representative of the lipid accumulation and should thus be used for quantification. For proteins quantification, both 1,548 or 1,655 cm^– 1^ can be used. Each of the five wavenumbers 1,043, 1,074, 1,107, 1,142, or 1,156 cm^– 1^ can be used for a correct quantification of carbohydrates.

### Application of Fourier Transform Infrared Spectroscopy Analysis to Infer Differences in Metabolites Quantifications Between Ecotypes of *P. tricornutum* During a N-Starvation Experiment

**FIGURE 6:**
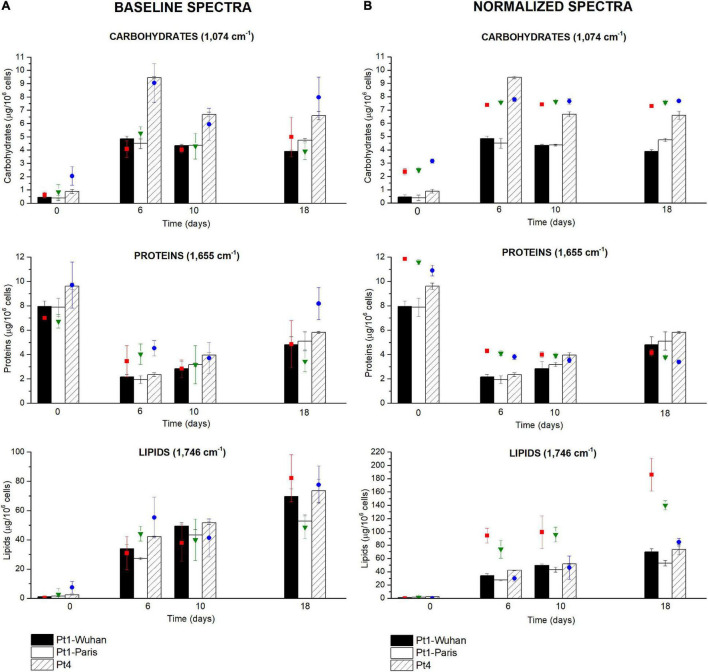
Carbohydrates, proteins and lipids quantifications through the FTIR based methodology performed during a N-starvation of 18 days in the different ecotypes and strains of *P. tricornutum*. Quantifications performed with standard biochemical protocols are presented as histograms in the graph while quantifications using FTIR absorbance are represented as colored-dots (red square, green triangle, and blue dots), considering **(A)** baseline-corrected spectra or **(B)** baseline and normalized-corrected spectra. FTIR quantifications are performed at 1,074 cm^– 1^ for carbohydrates, 1,655 cm^– 1^ for proteins, and 1,746 cm^– 1^ for lipids.

Around the world, ten different *P. tricornutum* ecotypes have been isolated and characterized. Being different at both genotypic and phenotypic levels, the ecotypes may also diverge in their cellular quota in major organic macromolecules (Martino et al., 2007; Rastogi et al., 2020). The described FTIR methodology was applied to characterize quantitatively the biochemical changes during 18 days of N-starvation exposure on two different ecotypes of *P. tricornutum i.e.* Pt1 and Pt4 with the aim to describe the potential metabolic divergences between them. Figure 6 compares the different cellular quota determined using conventional biochemical quantifications (bars) with those obtained using FTIR measurements (colored-dots, carbohydrates: 1,074 cm^−1^, proteins: 1,655 cm^−1^, lipids: 1,746 cm^−1^) after baseline correction (Figure 6A) and normalization (Figure 6B) using equations from Table 2.

Surprisingly, performing the normalization on spectra was not suitable for the comparison of metabolite contents between *P. tricornutum* ecotypes. The global trends of the different metabolites were similar (increase in carbohydrate and lipid contents and a decrease in protein content) but the differences between ecotypes were totally masked, indicating that the quantification using the SNV normalization may not be suitable for comparing different microalgal species or ecotypes unless specific regression equations to each of them are applied. Much better correlations are found when spectra are only baseline-corrected, indicating that this data treatment may be used to compare different species or ecotypes, keeping in mind the greater variability of the data when spectra are not normalized.

### Phenotypic Divergence Between *Phaeodactylum tricornutum* Ecotypes and Strains

**FIGURE 7:**
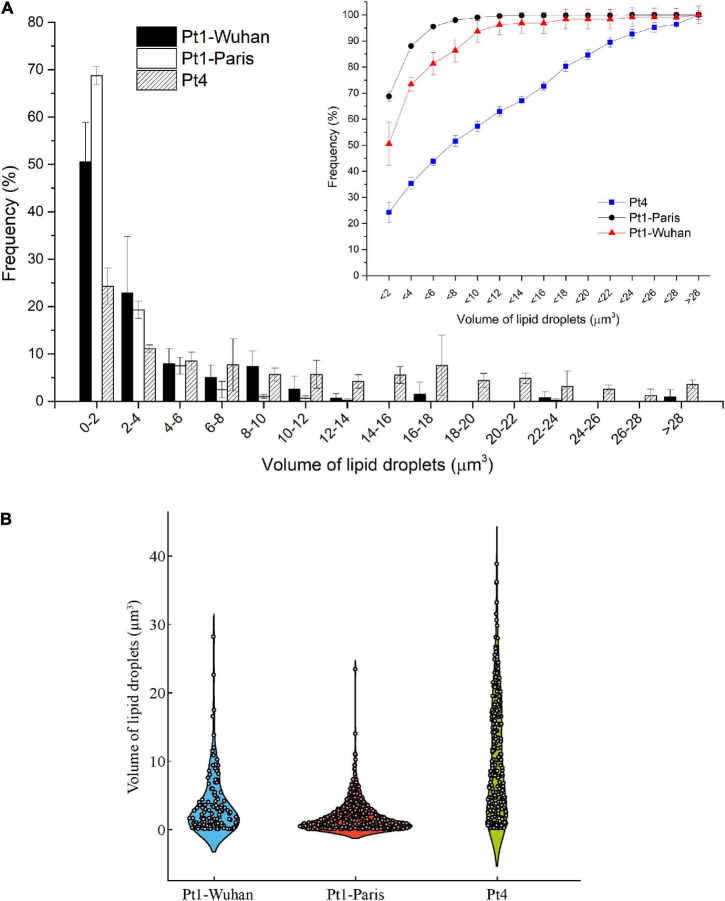
Volume analysis of LDs in the different ecotypes and strains of *P. tricornutum* after 18 days of N-starvation. **(A)** Histogram reporting the occurrence frequencies for LDs of specific dimension ranges. Line graph reports the cumulative occurrence frequencies for increasing size of LDs; **(B)** Violin plot reporting the occurrence frequency distribution of the different size of LDs.

Intrinsic differences between the two ecotypes can be observed in Figure 6A. Indeed, Pt4 shows a significantly higher Q_*Carbohydrates*_ compared to both Pt1 all along the N-starvation: at day 6 of N-starvation, Pt4 contains twice as much Q_*Carbohydrates*_ than both Pt1. The Q_*Proteins*_ is also slightly higher, though not significant, for Pt4, especially in N-repleted condition (Figure 6A). No matter the ecotype considered, the Q_*Lipids*_ increased along the starvation, reflecting the reorganization of the carbon flux toward the production of storage lipids (*e.g.* Hockin et al., 2012; Levitan et al., 2015; McCarthy et al., 2017). The Q_*Lipids*_ of Pt4 18 days after the removal of N supply is significantly higher than that of Pt1-Paris. This result is confirmed by both FTIR spectroscopy and biochemical quantifications and strengthened by confocal microscopic analyses performed using BODIPY 505/515. Indeed, the distribution of LDs 10 days after the removal of N supply (Figure 7) indicates that LDs accumulated by Pt1-Paris are globally weaker than Pt1-Wuhan themselves smaller than Pt4. While Pt1-Paris seems to possess a single population of small LDs (<2 μm^3^), Pt4 possesses two to three subpopulations in the culture: the first one in the lowest ranges (<2 μm^3^) and the second and third ones peaking around 6–8 and 16–18 μm^3^ (Figure 7A). This result is in line with the observations of Wong and Franz (2013) who found that Pt4 LDs ranged from 0.1 to 38 μm^3^ in N-deficient conditions. Our results also tend to confirm what suggested Jaussaud et al. (2020) and Leyland et al. (2020) using epifluorescence microscopy. Indeed, Leyland et al. (2020) observed a higher yield of LDs from cells disrupted by hypotonic shock in Pt4 compared to Pt1 with multiple small LDs for Pt1 and two relatively large LDs for Pt4. By analyzing the dynamics of LD formation in Pt1, Jaussaud et al. (2020) highlighted the stepwise generation of subpopulations, at least three, marked by an increase in size over time reaching a maximum value. Here, we confirm the existence of LDs subpopulations but also the presence of larger LDs for Pt4 (Figure 7B). Jaussaud et al. (2020) suggested that LDs grow until they reach a size limited by cell packing with other membrane organelles and by the stiffness of the limiting cell wall. They also observed larger LDs in a mutant line having larger cells compared to wild type cells. The presence of larger LDs in Pt4 could be due to larger Pt4 cells compared to Pt1. Indeed, cell measurements performed on brightfield microscope pictures showed differences in cell dimensions between the different ecotypes 18 days after the removal of N supply (Supplementary Figure 11). In particular, Pt4 ecotype appears 1.5 times wider than Pt1 ecotypes while small or no differences are observed in length.

**FIGURE 8:**
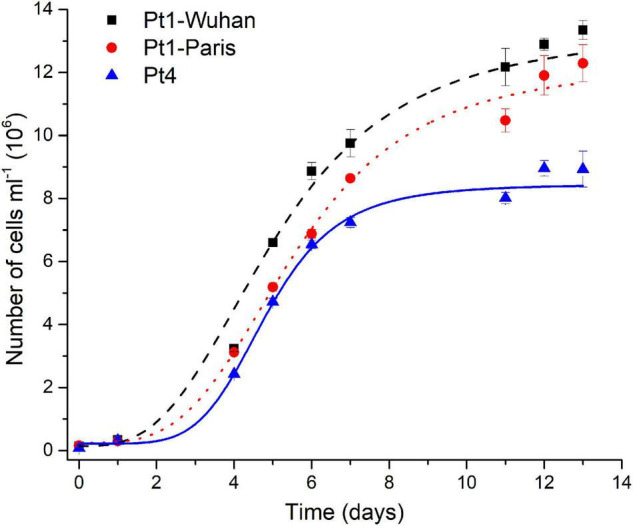
Growth curves of the different ecotypes and strains of *P. tricornutum* in N-replete condition. A logistic model was used for the determination of growth rates.

Pigment quantifications assessed (Supplementary Figure 12), revealed a higher total carotenoid (mainly represented by fucoxanthin) content and a lower Chl *a*/Chl c ratio in Pt4 than in Pt1, especially in N-repleted condition. According to Lamote et al. (2003), the lower values of the Chl *a*/Chl c ratio suggests a larger light harvesting antenna in Pt4 than in Pt1. This interpretation fits with the higher content in fucoxanthin in Pt4 since it is the main pigment of the fucoxanthin–protein complexes forming the light-harvesting antenna in diatoms (for a review see Büchel, 2020). This result is in line with the lower non-photochemical quenching capacity observed for Pt4 and proposed to be an adaptive trait to low light conditions (Bailleul et al., 2010). This accession has also been proposed to establish an upregulation of a peculiar light harvesting protein LHCX4 in extended dark conditions (Bailleul et al., 2010; Taddei et al., 2016). In N-repleted condition, a significantly higher maximal division rate was observed for Pt4 (0.99 ± 0.05 day^– 1^, *p* < 0.05) compared to Pt1-Wuhan (0.73 ± 0.06 day^– 1^) and Pt1-Paris (0.67 ± 0.05 day^– 1^) but also a lower maximal cell density for Pt4 (8 × 10^6^ of cells ml^–1^ while being around 11 and 12 × 10^6^ of cells ml^– 1^ for respectively Pt1-Paris and Pt1-Wuhan) (Figure 8). These results highlight a faster division rate and a higher N intake cell^−1^ for Pt4 compared to both Pt1 strains. In line with these observations, Rastogi et al. (2020) pointed out the presence of a higher copy number in the genome of Pt4 of a gene involved in nitrate assimilation (Phatr3_EG02286 encoding a nitrite reductase), suggesting an altered mode of nutrient acquisition in Pt4 compared to the other ecotypes.

Altogether, our results highlight that *P. tricornutum* ecotypes differ in their major macromolecules cell quotas and nutrient acquisition intensity: Pt4 cells absorb more nitrate and funnel more energy in the photosynthetic apparatus with consequently a higher ATP production and a faster division in N-repleted conditions. When facing N-starvation, Pt4 cells have a higher protein and carbohydrates content coupled with bigger cytoplasmic LDs, probably related to their larger cell volume compared to Pt1 cells. These results strengthen the genetic divergence already highlighted between Pt1 and Pt4. Indeed, using whole genome sequencing, Rastogi et al. (2020) draw a comprehensive landscape of the genomic diversity between the 10 accessions of *P. tricornutum*. They described strong genetic subdivisions of the accessions into four genetic clades with populations of each clade possessing a conserved genetic and functional makeup, likely a consequence of the limited dispersal of *P. tricornutum* in the open ocean. They observed that Pt4 was the most genetically divergent ecotype. However, the link between genetic and phenotypic divergence still remains to be elucidated.

Interestingly, the two Pt1 lines differ in their lipid content (Figures 6, 7). Pt1-Wuhan seems to possess, in addition to the small population of LDs present in Pt1-Paris (<2 μm^3^), a second subpopulation of LDs of higher volumes (8–10 μm^3^) (Figures 7A,B). This finding strengthens the significantly higher accumulation of neutral lipids measured using Nile red fluorescence in Pt1-Wuhan compared to Pt1-Paris. Although both lines originated from the same Culture Collection (CCMP 2561), this result may highlight a different evolutionary adaptation of the two strains induced by the culture conditions under which both Pt1 strains were maintained. As reported by Lakeman et al. (2009), in laboratory cultures, because of rapid growth rates and high population densities, mutations affecting the phenotype are likely to arise. Different processes can lead to genetic evolution in strains maintained in different laboratories. Genetic drift is likely to occur when serial transfer of microalgae are performed for maintenance: a relatively small and random inoculum of the genetic diversity is taken from the parent culture to start a new batch, introducing potentially genetic drift into the evolutionary history of the strain (Lakeman et al., 2009). Selective pressure may also be exerted by culture conditions. It occurs every time culture conditions are altered (temperature, irradiance, salinity, nutrients, etc.) and can be as subtle as a shift in light quality when growth chamber bulbs are replaced or as profound as a modified growth medium when a strain is sent to a new laboratory. The more times a strain is transferred between laboratories and subjected to novel growth conditions, the greater the potential for it to evolve properties that deviate from those of its original phenotype (Lakeman et al., 2009).

## Conclusion

Fourier transform infrared spectroscopy methodology developed in this study is suitable for the quantification of cellular quota of the major metabolites classes of unicellular organisms provided some simple rules are followed (Table 3): (1) deposit the adequate amount of cells, (2) performing baseline correction on spectra collected at 800–200 cm^– 1^, (3) performing the SNV normalization because it contributes to decrease the variability between biological samples, and (4) choose the adequate wavenumbers for biomolecule quantifications. In comparison to conventional chemical analyses, FTIR spectroscopy has striking advantages: (1) a high reliability and sensitivity, (2) a high speed of measurement procedure, (3) a low volume of sample is necessary, (4) no need to perform complex and time-consuming extractions, and (5) no need to determine the sample cell density which is time-consuming and subjected to error-prone. Data reported in this paper also confirm the metabolic divergence between Pt1 and Pt4 and even highlight phenotypic divergence between strains of the same ecotype cultured in laboratories with probably different maintenance modes. This suggests that exploring the intra-species processes of evolution can be trickier than expected and that even in our controlled laboratory environments, evolution do not cease to exert its influence.

**TABLE 3:** Rules to follow for a suitable quantification of lipids, proteins and carbohydrates in *P. tricornutum* through the described FTIR technique.

Feature	Parameter
FTIR modus	Transmission
Parameters	4 cm^– 1^ resolution, 32 scans/spectrum
Number of cells to deposit on the silica plate	1×10^6^ to 3×10^6^ cells
Number of biological replicates	3
Number of technical replicates	8
Collection of the raw spectrum	800–2,000 cm^– 1^
Baseline-correction	Rubber-band
Normalization	Standard normal variate
Lipids quantification	1,746 cm^– 1^
Proteins quantification	1,548 or 1,655 cm^– 1^
Carbohydrates quantification	1,043, 1,074, 1,107, 1,142, or 1,156 cm^– 1^

## Data Availability Statement

The raw data supporting the conclusions of this article will be made available by the authors, without undue reservation.

## Author Contributions

BS, JM, and MS: conceptualization, data curation, and initial writing. BS, JM, MS, AT, and MB: formal analysis. BS and JM: funding acquisition, project administration and supervision. BS, BV, JM, MS, and AT: investigation. BS, BV, JM, MS, FA, FL, FN, and AT: methodology. All authors contributed to manuscript revision, read, and approved the submitted version.

## Conflict of Interest

The authors declare that the research was conducted in the absence of any commercial or financial relationships that could be construed as a potential conflict of interest.

## Publisher’s Note

All claims expressed in this article are solely those of the authors and do not necessarily represent those of their affiliated organizations, or those of the publisher, the editors and the reviewers. Any product that may be evaluated in this article, or claim that may be made by its manufacturer, is not guaranteed or endorsed by the publisher.

## Footnotes

1
https://www.curveexpert.net/

2
https://github.com/r-hyperspec/hyperSpec

3
https://rdrr.io/cran/prospectr/

4
https://imagej.net/
